# Axial Flow Percutaneous Ventricular Assist Devices: Physiology, Procedural Considerations, and Clinical Applications—A State-of-the-Art Review

**DOI:** 10.1016/j.shj.2025.100773

**Published:** 2025-12-01

**Authors:** Ovidio De Filippo, Ilaria Pagliassotto, Valentina Braia, Riccardo Improta, Gianluca Di Pietro, Pierre Meynet, Martina Pecoraro, Maria Luisa Carbone, Marco Nebiolo, Francesco Bruno, Federico Giacobbe, Gaetano Maria De Ferrari, Antonio Loforte, Wojciech Wańha, Mauro Rinaldi, Fabrizio D’Ascenzo, Roberto Lorusso

**Affiliations:** a Division of Cardiology, Cardiovascular and Thoracic Department, Città della Salute e della Scienza Hospital, Turin, Italy; b Cardio-Thoracic Surgery Department, Maastricht University Medical Center+ and Cardiovascular Research Institute Maastricht (CARIM), Maastricht, The Netherlands; c Department of Medical Sciences, University of Turin, Turin, Italy; d Department of Clinical, Internal, Anesthesiological and Cardiovascular Sciences, Umberto I Hospital, La Sapienza University of Rome, Rome, Italy; e Department of Surgical Sciences, University of Turin, Turin, Italy; f Department Cardiology and Structural Heart Diseases, Medical University of Silesia, Katowice, Poland

**Keywords:** Cardiogenic shock (CS), high-risk percutaneous coronary intervention (PCI), Narrative review, Percutaneous mechanical circulatory support (pMCS), Percutaneous microaxial flow plumps (pMFP)

## Abstract

Percutaneous microaxial flow pumps have become an important form of temporary mechanical circulatory support for patients with advanced cardiac dysfunction, particularly in cardiogenic shock and high-risk percutaneous coronary intervention. By actively unloading the ventricle, reducing end-diastolic pressure and myocardial oxygen demand, and supporting systemic and coronary perfusion, Impella devices offer distinct physiological advantages over passive support strategies. This state-of-the-art review summarizes the mechanisms of ventricular unloading, device characteristics, procedural considerations, monitoring requirements, complication management, and contemporary clinical applications of axial-flow percutaneous ventricular assist devices. Current evidence suggests that, in selected patients with infarct-related cardiogenic shock, early and protocolized Impella use may improve survival, although this benefit must be balanced against higher rates of bleeding, hemolysis, vascular injury, thrombosis, and device-related complications. In high-risk percutaneous coronary intervention, Impella may enhance procedural stability and facilitate more complete revascularization, but a definitive survival benefit remains unproven. Emerging applications include right ventricular support, biventricular support, postcardiotomy shock prevention, ventricular tachycardia ablation, and combined use with venoarterial extracorporeal membrane oxygenation. Despite expanding use, major uncertainties remain regarding optimal timing, patient selection, standardized anticoagulation and weaning protocols, cost-effectiveness, and generalizability beyond infarct-related shock. Further randomized trials, registry-based analyses, and technological refinements are needed to define which patients benefit most while minimizing complications.

## Highlights

• Impella microaxial flow pumps enable active left ventricular unloading, reducing end-diastolic pressure, and myocardial workload while improving coronary perfusion and oxygen balance.
• Principal indications include infarct-related cardiogenic shock (SCAI C–D), where selected studies suggest potential mortality benefit and high-risk PCI, where devices enhance hemodynamic stability and completeness of revascularization, although a survival advantage remains unproven.
• Expanding applications include combined strategies such as ECMELLA (Impella + VA-ECMO) for LV unloading and BiPella for biventricular support in advanced heart failure.
• The Impella RP provides dedicated percutaneous right ventricular support and plays a key role in managing acute right heart failure in complex clinical scenarios.
• Clinical use is limited by costs, procedural complexity, and complications (vascular injury/bleeding, hemolysis, and pump thrombosis), with ongoing uncertainty regarding optimal timing, patient selection, and broader applicability.

## Introduction

Mechanical circulatory support (MCS) has evolved significantly over the past decades, providing crucial hemodynamic stabilization for patients with severe cardiac dysfunction.^1^ Several MCS strategies are currently available, each with distinct mechanisms and clinical applications. Intra-aortic balloon pumps (IAoBPs), one of the earliest forms of support, function by inflating and deflating in sync with the cardiac cycle to augment coronary perfusion and reduce afterload.^2^^,^^3^ However, their ability to enhance cardiac output (CO) is limited.^4^ Venoarterial (VA) extracorporeal membrane oxygenation (ECMO) (VA-ECMO) provides full cardiopulmonary support by oxygenating blood externally and delivering it back into the arterial circulation, making it highly effective for profound cardiogenic shock (CS) or cardiac arrest.^5^^,^^6^ However, ECMO increases left ventricular afterload, which may lead to ventricular distension and worsening pulmonary congestion if left unmanaged.^7^^,^^8^

In contrast, percutaneous ventricular assist devices (pVADs), particularly axial-flow pumps, have gained widespread adoption due to their ability to provide continuous forward flow while directly unloading the left ventricle (LV).^9^^,^^10^ Unlike IAoBPs, which rely on passive augmentation, or VA-ECMO, which bypasses the heart and lungs, axial-flow pVADs actively draw blood from the LV and propel it into the ascending aorta, reducing myocardial workload while maintaining systemic perfusion.^11^ This targeted unloading reduces left ventricular end-diastolic pressure (EDP), lowers myocardial oxygen demand, and improves coronary perfusion, potentially limiting infarct size in acute coronary syndromes and improving hemodynamic stability in high-risk procedures.^12^^,^^13^

The integration of these devices into clinical practice has transformed the management of various cardiovascular conditions, including acute myocardial infarction (AMI) with CS (AMI-CS), high-risk percutaneous coronary interventions (PCIs), and acute decompensated heart failure (ADHF).^14^^,^^15^ Recent trials and registry data have further defined their role by highlighting both their advantages and limitations in different clinical scenarios.^11^^,^^16^

Providing an in-depth overview of the physiological principles, mechanisms of action, and contemporary clinical applications of axial-flow pVADs, this review is intended as a comprehensive and practical guide for cardiologists and other clinical practitioners. In light of the expanding body of evidence and technological advancements surrounding Impella devices, this article integrates the latest evidence with practical clinical insights, critically appraises unresolved issues, explores future applications, and seeks to bridge the gap between theory and bedside practice.

Consistent with the Sex and Gender Equity in Research (SAGER) recommendations, we noted sex-disaggregated findings when reported.^17^ Given the narrative and targeted nature of this review, only a qualitative synthesis was provided.

## Physiology of Hemodynamic Support

### Mechanism of Ventricular Unloading

**Figure 1:**
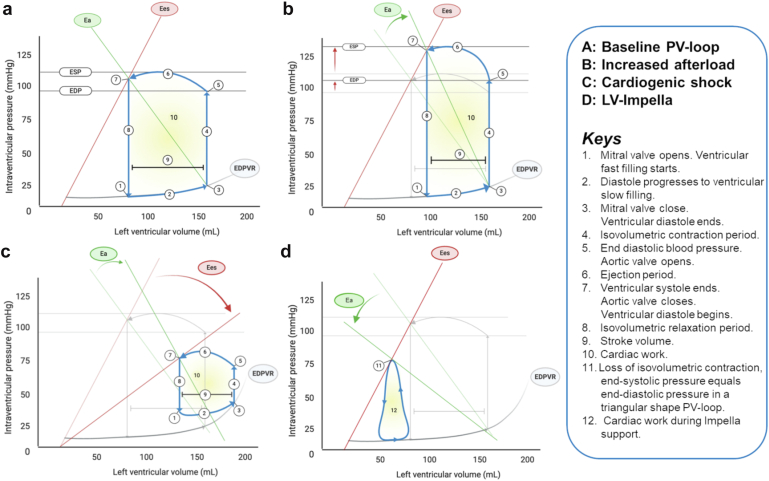
Schematic representation of the physiology of cardiac work. Panel (a) depicts baseline conditions, Panel (b) shows pressure-volume loops during increased afterload, Panel (c) illustrates cardiac work during cardiogenic shock, and Panel (d) demonstrates cardiac work with a left-sided microaxial flow pump in place. Abbreviations: Ea, arterial load; EDP, end-diastolic pressure; EDPVR, end-diastolic pressure-volume relationship; Ees, end-systolic elastance; ESP, end-systolic pressure; PV, pressure-volume.

Ventricular unloading is defined as the process of reducing myocardial work and oxygen consumption by decreasing the forces that the heart must exert during the cardiac cycle. The heart’s “load” is derived from time-varying forces that act upon the myocardial surface. The work performed by the heart to pump blood is determined by the pressure and volume dynamics of the cardiac cycle, known as the pressure-volume (PV) loop, first described by Suga^18^ and Sunagawa.^19^ This loop provides a comprehensive explanation of the changes in ventricular pressure and volume throughout the cardiac cycle (see Figure 1).

In a healthy heart, during diastole, blood flows from the left atrium to the LV at low pressure. During isovolumetric contraction, the LV remains constant in size while its pressure rises until it exceeds aortic pressure, causing the aortic valve to open and blood to be ejected. The sequence culminates in isovolumetric relaxation, which is succeeded by the opening of the mitral valve, thus initiating the subsequent cycle. The term “stroke volume” (SV) is defined as the difference between the volumes of blood at the end of diastole and the end of systole, representing the amount of blood ejected with each heartbeat. The area of the PV loop corresponds to the myocardial work performed to eject blood into the aorta (Figure 1a).

Preload, which affects ventricular filling, is indexed by end-diastolic volume or EDP. In conditions involving diastolic dysfunction, the relationship between filling pressure and volume changes, causing shifts in the PV loop either to the right or left.^1^^,^^20^

Sunagawa et al.^21^ defined afterload as “effective arterial elastance” (Ea), which incorporates extracardiac factors affecting ventricular ejection. An increase in afterload elevates the gradient of Ea along the end-systolic pressure (ESP)-volume relation, consequently shifting the PV loop to the right, resulting in an increase in ESP and a decrease in SV (Figure 1b). As Walley et al.^20^ explain, ventricular elastance interacts with Ea during ejection, so an increase in afterload raises ESP and reduces ejection, thus decreasing mechanical efficiency.

The interaction between ventricular ejection and arterial pressure changes defines ventriculo-arterial coupling, which reflects global cardiovascular performance and efficiency. This coupling is represented by the Ea/Ees (end-systolic elastance) ratio, where Ees indicates myocardial contractility and systolic stiffness, while Ea measures arterial load, including factors like vascular resistance and compliance. The optimal level of this coupling is achieved when the ratio is approximately 1, indicating that the work performed by the ventricle equals the total work done with the minimal energy expenditure.

Among the current strategies aimed at percutaneously unloading the LV, transvalvular percutaneous microaxial flow pumps (mAFPs) (represented by Impella) have emerged to primarily unload the LV while simultaneously augmenting CO.

The left-sided Impella device, which draws blood directly from the ventricle, reduces ventricular end-diastolic volume and EDP, shifting the PV loop to the left. The continuous pumping of blood from the LV to the aorta, regardless of the phase of the cardiac cycle, results in the loss of isovolumetric contraction and relaxation, leading to a reduction in stroke work. As a result, the PV loop assumes a triangular shape, reducing myocardial wall tension and workload (see Figure 1d). Both effects reduce myocardial work and myocardial oxygen demand.^22^, ^23^, ^24^

LV unloading triggers biological responses that depend on the underlying cause of LV overload and the clinical setting.^25^

In chronic overload, changes in LV pressure and volume impair cardiac energy metabolism, reducing adenosine triphosphate production and utilization. Mechanical unloading can reverse these changes by activating cardioprotective pathways and improving metabolic efficiency, including increased glycogenolysis, glycogen utilization, and acylcarnitine levels.^26^^,^^27^ It also restores gene expression of PGC-1α, a key regulator of mitochondrial function, leading to an overall improvement in substrate utilization and energy production.^28^ In addition, as demonstrated in animal models, unloading can reverse pathological LV remodeling, including hypertrophy, fibrosis, and changes in autophagy, suggesting the potential to reverse these detrimental structural changes by reducing metalloproteinase levels and tissue inhibitors of metalloproteinase expression.^29^

In ischemic hearts, chemokines such as stromal-derived factor 1 alpha are upregulated, promoting apoptosis and tissue remodeling, mediating the ischemia-reperfusion injury that leads to the development of heart failure (HF) after AMI. Several preclinical studies suggest that cardioprotective strategies, such as primary unloading,^12^^,^^25^ may help to reverse this damage. Unloading has been shown to preserve stromal-derived factor 1 alpha levels, reduce pro-apoptotic signaling, and reduce infarct size, thereby improving outcomes in AMI.^12^

Mechanical LV unloading during the acute phase of myocardial infarction reduces infarct size, which is strongly associated with all-cause mortality and HF hospitalizations.^30^ The reduction in infarct size is due to a decrease in myocardial oxygen consumption: by reducing LV EDP and increasing LV Ees, LV unloading reduces stroke work, a major determinant of oxygen consumption.^1^^,^^12^ In addition, LV unloading activates protective pathways, including the extracellular signal-regulated kinase (ERK) and protein kinase B (Akt) pathways, leading to a reduction in cellular apoptosis signaling.^13^

### Impacts on Myocardial Oxygen Supply/Demand Balance

The hemodynamic effects of left-sided Impella devices have been examined in several observational studies. These effects can be summarized as follows:

1. The device functions by drawing blood directly from the LV, thereby ensuring active unloading and reducing LV EDP and volume. This, in turn, decreases LV work and myocardial wall tension, leading to a reduction in myocardial oxygen demand.
2. Left ventricular unloading results in decreased pulmonary capillary wedge pressure and secondary reduction in right ventricular afterload.^10^
3. Due to the continuous pumping of blood in the aortic root, Impella provides active flow, thereby increasing mean arterial pressure (MAP), diastolic pressure, CO, and cardiac power output (CPO).^30^, ^31^, ^32^ Impella is expected to improve peripheral organ perfusion by increasing MAP and blood flow.^33^
4. The synergistic effect of increased mean aortic pressure and decreased myocardial wall tension may increase coronary blood flow by reducing compression of the microvasculature, enlarging the lumen diameter, and reducing resistance to flow, thereby improving myocardial oxygen supply.^34^^,^^35^

Impella increases blood flow and aortic pressure, resulting in an increase in CO and mean aortic pressure. The actively generated forward flow depends on (a) the specific device, (b) the performance “P” level setting, and (c) the pressure gradient across the aortic valve. Higher “P” levels or a lower pressure gradient result in increased forward flow. The increase in systemic CO results from the net effect of native CO reduction after ventricular unloading and the forward flow contribution of the Impella pump.^36^

Mechanical oxygen demand is determined by 2 elements: (a) the amount of mechanical work the muscle produces and (b) the amount of myocardial potential energy, which is related to wall tension.^37^ By actively drawing blood from the LV to the aortic root, Impella reduces total filling volume and pressure, leading to a reduction in SV. According to the Frank-Starling mechanism, if the heart fills less, it expands less and reduces its stroke output, which corresponds to a reduction in its mechanical work.

The myocardial potential energy is related to the amount of wall tension stored in the muscle, which is related to end-diastolic volume and EDP, as described by the Law of LaPlace. The reduction in these parameters leads to reduced microvascular compression and increased myocardial perfusion, with increased oxygen supply. In addition, the active unloading results in reduced wall tension, directly reducing myocardial oxygen demand.

The net result is a decrease in myocardial oxygen demand with a concomitant increase in oxygen supply through an increase in coronary blood flow. This result has been analyzed in several animal models: Reesink et al.^36^ approximated the demand-supply balance as the ratio of mechanical work to the coronary flow, demonstrating a 36% improvement with Impella compared to an 18% improvement with IAoBP, with Impella benefit more pronounced at lower native CO. Sauren et al.^34^ also investigated the net change in oxygen demand-supply balance, including the potential energy component: analyzing the ratio using the PV area and the coronary flow, they reported a 69% improvement with Impella compared to a 15% improvement with IAoBP alone. Notably, both of these studies employed an Impella 5.0 device operating 3.8 and 4.2 L/min, respectively.

### Specific Hemodynamic Effects (Coronary Flow Augmentation)

The Impella has been suggested to increase coronary artery flow, a parameter determined by the interplay between MAP and microvascular resistance. By reducing peak wall stress and microvascular resistance, it potentially enhances coronary blood flow even in patients with reduced ejection fraction.^35^

In addition, the continuous flow generated by Impella may provide a more sustained support of diastolic coronary perfusion. This differs from the IAoBP, which augments flow transiently during diastolic inflation but rapidly loses effect before systole.

Experimental data from Sakata et al. in porcine models further suggested 3 mechanisms by which Impella may improve coronary blood flow:

1. Maintaining high diastolic pressure in the aortic root after aortic valve closure, thereby increasing coronary driving pressure.
2. Reducing left ventricular EDP and wall tension, which decreases extravascular compression of the microcirculation and lowers microvascular resistance.
3. Unexpectedly, prolonging the diastolic phase and thus extending the duration of elevated coronary driving pressure.^38^

Importantly, while these findings provide important mechanistic insights, they derive mainly from animal models, and their direct translation into the clinical setting remains uncertain.

### Differences in Physiology for Left- vs. Right-Sided Support

The optimal right ventricle (RV) function results from a complex interplay of several factors, including pulmonary artery (PA) pressures (afterload), venous return (preload), right ventricular myocardial contractility, pericardial compliance, and interventricular dependence.^39^^,^^40^ Any disruption in these physiological parameters can result in RV failure,^41^ with signs and symptoms of right ventricular dysfunction.^42^^,^^43^

Compared with the LV, the RV has thinner walls^44^ and pumps blood into a low-resistance pulmonary circulation, making it less able to tolerate sudden increases in afterload.^45^ In both chronic and acute settings, a small increase in afterload can cause a large reduction in SV without an increase in pressure generation.^39^^,^^46^ Volume overload, occurring because of increased venous return, can lead to progressive RV dilatation and dysfunction (e.g., left ventricular assist device [LVAD] implantation or acute LV failure). Myocardial damage reduces RV contractility and SV, which can precipitate the LV impingement due to interventricular dependence. In addition, the performance of the RV is closely linked to the pericardium characteristics, and its alterations can impair the normal RV function. Furthermore, RV failure can also occur as a potentially life-threatening complication following cardiac surgery.^46^

The right-sided device (Impella RP) employs an mAFP with an inflow tract in the inferior vena cava and an outflow tract in the PA. It functions as a direct RV bypass, actively unloading the RV, reducing RV workload and systemic venous congestion, and increasing PA flow. At maximal speed (33,000 rpm), it delivers up to 4.0 L/min of flow.^47^ This results in increased mean PA pressure, enhanced LV preload, and elevated CO, assuming preserved or assisted LV function.^47^^,^^48^

Device performance depends on the pressure gradient between the PA and right atrium (pressure head): in severe RV dysfunction, a low-pressure head yields higher flow, whereas in pulmonary hypertension, elevated PA pressure limits flow at a given speed.^49^ By augmenting PA flow, Impella RP increases LV preload and, when LV function is preserved, improves CO and end-organ perfusion.^49^, ^50^, ^51^ In LV dysfunction, however, increased PA flow may raise pulmonary capillary wedge pressure and LV afterload.

As demonstrated in clinical and preclinical models of RV failure, Impella RP reduces RV end-diastolic volume and EDP, decreases myocardial workload and oxygen consumption,^44^^,^^45^ and enhances LV performance through improved preload and ventricular interdependence, leading to an overall increase in biventricular pressure–volume area.^49^^,^^52^^,^^53^

**Figure 2:**
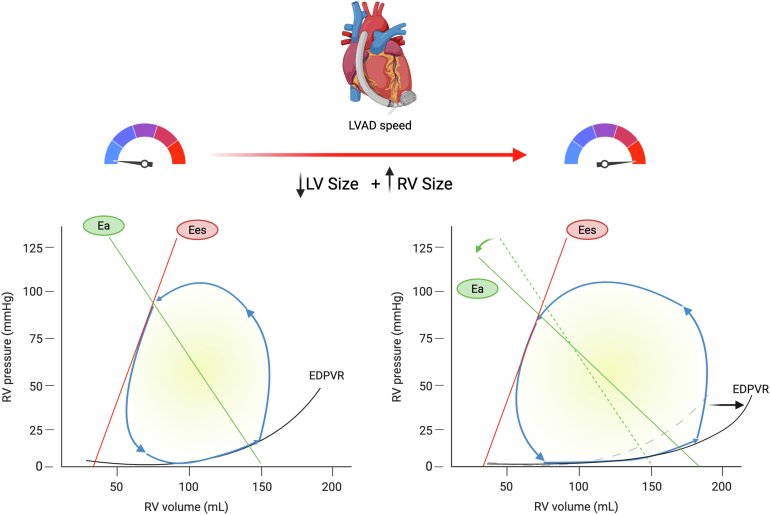
Schematic representation of right ventricular pressure-volume relationship during LVAD support. As LVAD speed rises, left ventricular size decreases while right ventricular size increases. The right ventricular EDPVR shifts rightward, reflecting increased compliance. In contrast, the end-systolic pressure-volume relationship remains relatively unchanged, indicating preserved systolic function. Right ventricular stroke volume increases due to enhanced preload and decreased afterload, improving overall right ventricular performance. Abbreviations: Ea, arterial load; EDPVR, end-diastolic pressure-volume relationship; Ees, end-systolic elastance; LVAD, left ventricular assist device.

During LV pVAD support, enhanced LV output, although associated with greater RV volume, exerts minimal effects on RV filling pressures and reduces RV afterload and pressure–volume area (see Figure 2).^54^ In contrast to the hemodynamic profile after LVAD implantation—typically requiring pericardiectomy and cardiopulmonary bypass—Impella support promotes favorable RV adaptation and load reduction in parallel with LV unloading.^55^

## Types of Impella Devices

The Impella system is available in multiple configurations, differing in size, insertion technique (either percutaneous or surgical), and maximal flow capacity. Currently available devices include left-sided Impella models—Impella Cardiac Power (Impella CP), Impella 5.5, and Impella Expandable Cardiac Power (Impella ECP)—as well as a right-sided Impella variant (see Table 1). The first device introduced to support the LV, namely Impella 2.5, is no longer commercially available.

**Table 1:** Overview of Impella devices

	Maximum flow rate	Sheath size	Access	Placement	Maximum recommended duration of support
Impella 2.5∗	2.5 L/min	13 Fr	Percutaneous femoral artery or axillary artery (in cases where femoral access is not possible)	Retrograde insertion into the ascending aorta, across the aortic valve, and into the left ventricle	CS: up to 4 days
					HR-PCI: up to 6 hours
Impella CP (Cardiac Power)	3.7 L/min	14 Fr	Femoral or axillary artery	Similar to the Impella 2.5 with improved flow support	CS: up to 4 days
					HR-PCI up to 6 hours
Impella 5.5	5.5 L/min->up to 6.2 L/min	Requires surgical cut-down access	Axillary artery (surgical approach)	Advanced percutaneously or surgically into the left ventricle for prolonged circulatory support	Up to 14 days
Impella ECP (Expandable Cardiac Power)	Approximately 5 L/min	9 Fr expandable to 14 Fr	Percutaneous femoral artery	Designed to provide higher flow rates with a lower-profile insertion system	HR-PCI: up to 6 hours
Impella RP	Up to 4.4 L/min (commonly 4.0 L/min specified)	23 Fr	Percutaneous femoral vein (anterograde)	Advanced through the tricuspid and pulmonary valves into the pulmonary artery for right ventricular support	Up to 14 days

∗ This device is no longer commercially available. Abbreviations: CS, cardiogenic shock; HR-PCI, high-risk percutaneous coronary intervention.

Left-sided Impella devices function as microaxial pumps positioned across the aortic valve, with an inflow tract located within the LV and an outflow tract in the ascending aorta.

As described above, these devices provide continuous antegrade flow throughout the cardiac cycle, actively unload the LV, reduce myocardial workload, and improve overall perfusion.

Right ventricular failure poses distinct therapeutic challenges because dedicated percutaneous support devices for the RV are less widely available than those for the LV.^56^^,^^57^ The development and clinical integration of right-sided Impella technology aim to address this gap by providing targeted hemodynamic support for patients with right ventricular dysfunction.^46^ As described above, Impella RP, by actively bypassing the RV, actively unloads the RV, reduces RV workload, and increases PA flow.

The Impella RP was approved for RV support in 2015.^58^ The device received regulatory approval under a humanitarian device exemption, based on preliminary findings from the multicenter RECOVER RIGHT study—a prospective, nonrandomized, multicenter investigation designed to evaluate outcomes following the implantation of the Impella RP in patients with RV failure occurring within 48 hours of AMI, cardiac surgery, or LVAD placement.^56^ This trial demonstrated that the Impella RP is a safe, reliable, and effective intervention for improving hemodynamics in critically ill patients with limited therapeutic options and a poor prognosis.^47^

Current worldwide data on the use of different systems (CP, 5.5 systems, and right ventricular systems) are not systematically reported in the medical literature, and the references provided do not enumerate global or country-specific usage numbers for these categories. The global distribution of Impella devices is marked by widespread and expanding use in North America, Europe, and selected regions of Asia, although adoption rates vary considerably across countries and healthcare institutions. In contrast, use in other regions remains comparatively limited.

Some national registries, such as the Italian IMP-IT registry, along with collaborative European expert networks, have documented a notable rise in utilization for both CS and high-risk PCI indications.^59^ In Japan, the nationwide J-PVAD Registry demonstrated widespread use of Impella support in CS secondary to AMI.^60^ The adoption in other parts of Asia, Latin America, and the Middle East remains variable and less well documented. Among these devices, the Impella CP is the most extensively employed, primarily due to its suitability for percutaneous LV support in cases of CS and high-risk PCI.^61^ The worldwide use and distribution of Impella RP is predominantly concentrated in the United States with limited but growing adoption in select centers in Europe and other regions.

The lack of scientific data should prompt reflection on the need to collect data on the use of different Impella devices.

### Access and Characteristics

Percutaneous insertion for left-sided devices is achieved via a retrograde arterial approach: the catheter is introduced through the femoral artery (or the axillary artery when the femoral approach is not feasible due to peripheral artery disease [PAD]).^62^ The catheter is then advanced into the ascending aorta and across the aortic valve into the LV, guided by fluoroscopic and/or echocardiographic imaging. The Impella 2.5 could be placed using a 13 Fr sheath and was expected to provide a maximum flow rate of 2.5 L/min. The currently available Impella CP requires a 14 Fr sheath and delivers a maximum flow rate of 3.7 L/min.^57^ By contrast, the 21 Fr (Impella 5.5) device, with a maximal flow rate of 5.5 L/min, mandates a surgical approach for transfemoral, transaxillary, or trans-subclavian retrograde implantation. One possible advantage of the axillary approach is the potential for long-term support, enabling patient mobility.

Both the Impella CP and 5.5 are equipped with SmartAssist, which displays pump parameters and an estimated device position on the Automated Impella Controller. SmartAssist uses optical sensors to derive positional waveforms and provides calculated surrogates of left ventricular EDP, MAP, and CPO. These data can assist with monitoring, assessment of device position, and weaning but do not replace standard invasive hemodynamic assessment.^63^

The Impella RP catheter is placed via a percutaneous antegrade approach through the femoral vein and advanced across the tricuspid and pulmonary valves into the PA.^48^^,^^64^ It is introduced using a 22-Fr sheath under fluoroscopic guidance, advanced over a 0.025-inch platinum super-stiff wire, and provides a maximum flow rate of 4.0 L/min, enabling temporary RV support for up to 14 days.^46^ To enhance the deliverability of this device, the Impella RP has evolved into the Impella RP Flex with SmartAssist, which received FDA approval on March 31, 2023. This device has been designed to facilitate placement through a jugular venous approach, providing enhanced deliverability via a more flexible catheter. Similar to left-sided Impella devices, the “SmartAssist” technology incorporates dual-sensor monitoring to help guide optimal pump management in real time. Providing a flow of over 4.0 L/min, this device is approved for circulatory assistance of up to 14 days in patients with body surface area ≥1.5 m².

The Impella ECP device has not yet been approved for use in humans, although several clinical trials are currently underway, including the Early Feasibility Study (NCT04477603) and the pivotal ECP trial (NCT05334784).^61^^,^^65^ This self-expandable percutaneous LVAD comprises an extracorporeal handle with an integrated motor and the catheter itself. The cannula, containing both the inflow and outflow tracts, is situated at the distal portion of the catheter, includes a distal pigtail to secure stable positioning in the LV and prevent suction events, and delivers flows up to 3.9 L/min.^65^ Its primary advantage is the potential for insertion without a preplaced guidewire, thus reducing the required introducer sheath size (10 Fr) and minimizing insertion time as well as periods of low or no flow. Using only transesophageal echocardiography guidance, this feature could eventually extend implantation to noncatheterization laboratory settings.

### Indications

While several clinical scenarios call for the use of Impella devices, many of the current usage strategies are largely founded upon relatively sparse randomized controlled data.

The main indication is for patients with stages C–D Society for Cardiovascular Angiography & Interventions (SCAI) CS refractory to medical treatment, whether secondary to AMI, ADHF, or myocarditis, and for use as a bridge to recovery, heart transplantation, or a durable device.^66^ Pragmatic considerations and SCAI C–D cues supporting Impella use include refractory hypotension or hypoperfusion despite optimized care; escalating vasopressor/inotrope requirements or rising lactate/organ dysfunction; imminent PCI or bridge-to-recovery/decision with expected LV unloading benefit; feasible vascular access and no absolute contraindication (e.g., LV thrombus, mechanical aortic valve); assessment of RV failure with a plan for support if indicated; and availability of a multidisciplinary team to provide protocolized monitoring, anticoagulation, and weaning.

To date, no score reliably predicts benefit from microaxial support; existing tools mainly stratify mortality risk. The Intra-aortic Balloon Pump in Cardiogenic Shock II (IABP-SHOCK II) and CardShock scores (i.e., age, lactate, creatinine, etc.) can flag very high-risk AMI-CS phenotypes for multidisciplinary review, but they should not be used as automatic triggers for implantation.^67^^,^^68^ In DANGER-SHOCK, the treatment effect was consistent across subgroups with a possible greater effect at lower MAP, supporting selection by SCAI stage, lactate, and MAP rather than a single score.

A specific scenario that involves biventricular HF utilizes 2 microaxial flow Impella catheters—one for the left side (e.g., CP or 5.0) and another for the right side (Impella RP)—and represents a novel method of biventricular support in refractory CS. Although no large clinical trials have evaluated the effectiveness of BiPella (biventricular support), several case reports^69^^,^^70^ and small retrospective analyses suggest its feasibility, with reductions in cardiac filling pressures and improvements in CO across various causes of CS.^64^^,^^71^ However, evidence is scant and largely limited to case reports and small retrospective series, with heterogeneous outcomes and no randomized trials.

Another potential application of the Impella device is the prophylactic prevention of postcardiotomy cardiogenic shock (PCCS) in patients undergoing high-risk cardiac surgery. LV dysfunction constitutes a significant prognostic factor in cardiac surgery patients,^63^ and PCCS, occurring in approximately 2% to 6% of cases, is particularly common among those with reduced ejection fraction. Traditional management of postcardiotomy shock has relied on inotropes and vasopressors, often coupled with afterload reduction via IAoBP therapy. However, these strategies have shown variable efficacy and carry inherent risks. Preemptive MCS, such as an Impella device, may improve coronary perfusion, reduce myocardial oxygen demand, and decrease wall stress, thereby potentially enhancing postoperative outcomes. Ongoing investigations, including the IMpella-Protected cArdiaC surgery Trial (IMPACT Trial, NCT05529654), are exploring the role of prophylactic Impella deployment in this setting.

Hemodynamic support in high-risk PCI, determined by a heart team discussion, remains an area of clinical ambiguity due to the lack of systematic comparative evaluations of support devices for this indication. Patients with severely impaired LV function undergoing PCI for complex lesions (e.g., left main stenosis or 3-vessel disease) face a significantly higher mortality risk than the general PCI population.^9^ During PCI, contrast injections, balloon inflations, atherectomy passes, and stent placements temporarily disrupt blood flow to the target coronary artery, causing a transient negative inotropic effect. While typically tolerated, patients with markedly compromised LV function may decompensate rapidly, jeopardizing procedural success and overall outcomes. Impella support can help maintain hemodynamic stability and potentially allow for more complete revascularization. For high-risk PCI, a 2025 score derived in Impella-assisted cases may aid *procedural* risk counseling, but it still lacks adequate validation.^72^

Impella devices have also been used to facilitate ventricular tachycardia ablation, given that 50% to 80% of patients with structural heart disease referred for ventricular tachycardia ablation present with hemodynamically unstable VT.^73^^,^^74^ By maintaining end-organ and coronary perfusion during prolonged periods of mapping or ablation, Impella support may reduce the risk of hemodynamic collapse and improve procedural success.

Indications for Impella use vary with each device. The previously available Impella 2.5 and the current Impella CP are approved for patients with CS within 48 hours of AMI or open-heart surgery and for those with cardiomyopathy or LV failure not responding to medical therapy. These models are also cleared for high-risk PCI. Notably, the Impella 2.5, with a maximum flow of 2.5 L/min, could be deemed suboptimal in individuals with high body mass index (BMI) or profound shock who require greater flow support. Conversely, the Impella 5.5 is currently approved exclusively for patients with CS. The Impella RP supports patients experiencing CS secondary to acute primary RV failure and can be utilized independently or in conjunction with a left-sided device (BiPella).^66^

Crucially, evidence on sex-based outcomes with microaxial support is mixed, and sex-disaggregated outcomes are inconsistently reported, limiting formal synthesis of evidence by sex. A 2025 real-world analysis of AMI-CS found no mortality difference by sex but higher acute kidney injury (AKI) and readmissions in men, while women had more critical limb ischemia.^75^ Prior cohorts similarly showed no 30-day mortality difference between sexes with microaxial percutaneous LVADs, although broader CS literature reports underuse of MCS and worse outcomes in women, underscoring the need for equitable access and careful limb-risk mitigation. ^22^^,^^76^ Underreporting of sex-disaggregated outcomes constrains generalizability and precludes robust sex-stratified meta-analysis; future trials should prespecify sex-stratified endpoints and analytic plans per SAGER recommendations.

Similarly, future studies should prespecify age- and frailty-stratified analyses to clarify whether treatment effects vary across the age spectrum.

### Contraindications

#### Left-Sided Impella

Absolute contraindications for the use of left-sided Impella devices include the following:

• Presence of a left ventricular thrombus (risk of device malfunction or systemic embolization)
• Presence of a mechanical aortic valve
• Aortic dissection^77^

Relative contraindications include the following^63^:

• Severe aortic regurgitation (initially, severe aortic stenosis was also considered a contraindication, but in specific cases, the support provided by Impella CP or 5.5 may exceed native cardiac function despite additional narrowing caused by the device)
• Obstructive hypertrophic cardiomyopathy
• Hematologic disorders predisposing to hemolysis
• Ascending aortic aneurysm
• Significant PAD

#### Right-Sided Impella (Impella RP)

Contraindications for the right-sided Impella include the following^48^:

• Mechanical tricuspid or pulmonary valve
• Severe tricuspid valve stenosis
• Severe pulmonary valve stenosis or insufficiency
• Thrombosis in the vena cava, right atrium, RV, or PA

## Monitoring During and After Use

Monitoring is one of the pillars in the management of the critically ill patient, particularly when supported by a temporary mechanical circulatory support (tMCS). Monitoring, both in the cath lab and the intensive care unit, needs a structured workflow to rapidly detect any change in patient and tMCS status, intercept complications, and forecast subsequent management. Invasive hemodynamic and clinical monitoring, echocardiography, and specific tMCS parameters taken together can provide a comprehensive view of patient (and device) condition and guide subsequent management.

### Key Monitoring Parameters

Every patient supported with pVAD should, at a minimum, be monitored with an arterial line and a central venous catheter. On the arterial line, loss of pulsatility (LOP) may indicate device displacement or the presence of ventriculo-arterial uncoupling.^77^ Central venous catheter provides a direct measurement of patient RV preload and, together with arterial blood gas analysis, an indirect estimate of peripheral perfusion (i.e., central venous oxygen saturation).^78^, ^79^, ^80^, ^81^ Although routine practice may differ between centers, use of a pulmonary artery catheter (PAC) should be advised to assess preload, RV status, degree of LV unloading, total CO, vascular resistances, and peripheral perfusion.

**Figure 3:**
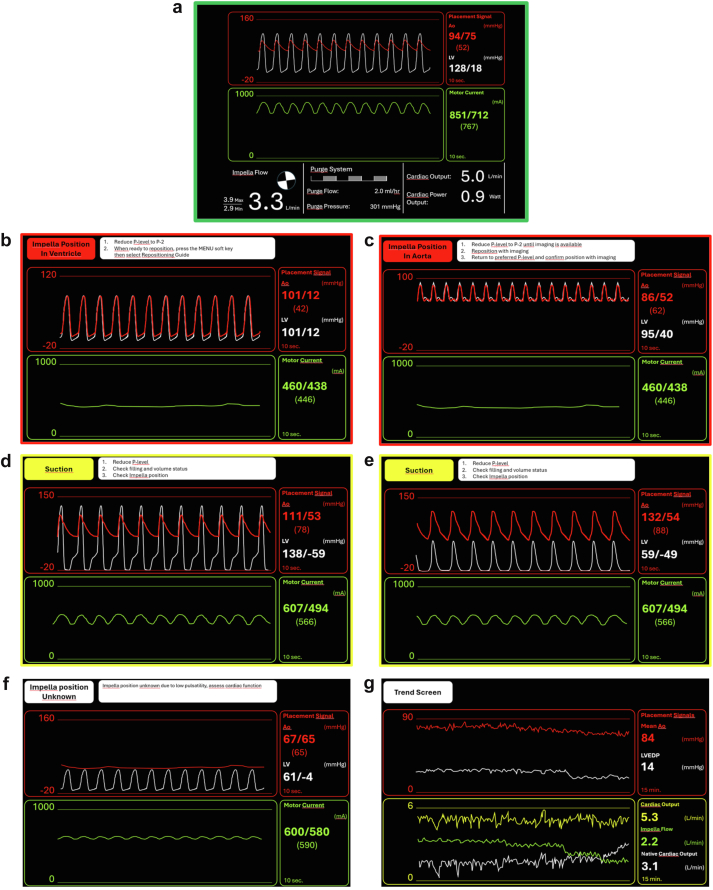
Example of Impella SmartAssist Waveforms. Panel (a) Correct left-sided Impella positioning and function. This panel illustrates 3 primary signals displayed by SmartAssist technology during left-sided Impella support. Motor current (green waveform) reflects pump flow and responds to changes in pressure gradient across the aortic valve, resulting in pulsatility. Placement signal (red waveform) serves as a surrogate of aortic pressure and can shift toward a ventricular pressure contour if the outlet crosses the aortic valve (malposition). Left ventricular pressure (white waveform) is derived by subtracting the aortic pressure head from the outflow side of the pump. A drop below −40 mmHg in diastole may trigger a suction alarm, suggesting low preload or malposition. Together, these signals guide optimal Impella positioning, flow adjustments, and the early detection of complications such as suction events, device malrotation, or abrupt reductions in native cardiac output. Panel (b) Ventricularization of aortic curve (red) and flattening of motor current curve (green), suggesting Impella malposition (ventricular dislocation). Panel (c) Loss of ventricular waveform in ventricular curve (white) and flattening of current motor curve (green), suggesting Impella malposition (aortic dislocation). Panel (d) and Panel (e) Alarms of intermittent and continuous suction, respectively. Panel (f) Current motor (green) and aortic curve (red) shows low pulsatility (likely due to very low cardiac function). SmartAssist system reveals impossibility to localize the device and suggests assessing cardiac function. Panel (g) Trend of curves relative to mean aortic pressure (red), left ventricular end diastolic pressure (white, above), total cardiac output (yellow), Impella flow (green), and native cardiac output (white, below).

SmartAssist function provides measured and derived parameters that are useful both for patient and device management (Figure 3). Three fundamental waveforms are displayed on the console^82^:

1. Motor current (*green waveform*), a measure of flow generated by the rotor (i.e., pump flow). This typically shows pulsatilty because of the variable pressure gradient as the device sits across the aortic valve. LOP may indicate a severe depression in native cardiac contractility or, more often, a displacement of the pVAD, with inlet and outlet sitting on the same side of the aortic valve;
2. Placement signal (*red waveform*), continuously measured by an optical sensor located at the outlet, that resembles an aortic pressure waveform. Ventricularization of placement signal indicates a displacement of the device in the ventricular cavity;
3. Left ventricular pressure (*white waveform*), calculated by subtracting from the aortic pressure the pressure head (i.e., pressure difference between outlet and inlet), the latter continuously derived from motor speed and current. Arterialization of this waveform indicates a displacement of the device in the aorta. When the left ventricular diastolic pressure falls below −40 mmHg, a suction alarm is activated. Diastolic suction may indicate insufficient LV preload, while continuous suction reflects a suboptimal pVAD position.^83^

Pump flow, directly related to the level of P (i.e., rotor speed), is continuously shown on the bottom left of the screen. Manually inserting a value of CO (by means of PAC or echocardiography), the Impella console can derive native CO and CPO, and the latter associated with short-term survival. In the Trend Screen, the parameters plotted against time are displayed, a function particularly useful in the setting of weaning.^84^^,^^85^

**Figure 4:**
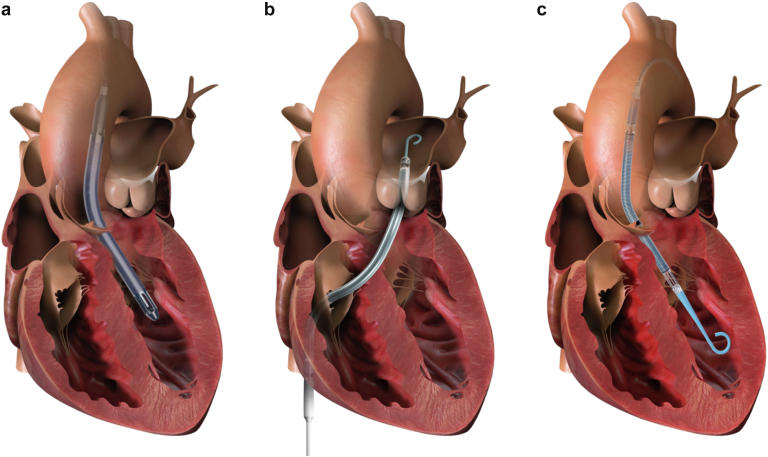
Simplified cartoons of Impella 5.5 (a), Impella RP (b), and Impella CP (c) in their proper positions. Published with permissions of Abiomed.

Bedside echocardiographic monitoring is a fundamental tool in monitoring Impella-supported patients. Besides its role before pump implantation, day-to-day echo assessment is imperative to evaluate proper pump positioning, assess LV and valve function, and assess RV status.^86^^,^^87^ Moreover, point-of-care ultrasound has an essential role in case of complications (see after). Accurate device positioning is essential to provide an adequate hemodynamic support and prevent complication occurrence.^86^ Per manufacturer instructions, for left-sided devices, the inlet opening should be positioned at 3.5 to 4 cm from the aortic valve, while the outlet should be placed in the ascending aorta. Malrotation, defined as an abnormal orientation of the inlet away from the LV apex and toward the LV lateral wall, has been associated with suboptimal LV unloading and worsened pulmonary and RV hemodynamics.^88^ Proper positioning should therefore be verified on a daily basis or whenever needed, with the aid of echocardiography and with analysis of the display waveform (see before). Figure 4 shows simplified cartoons of Impella 5.5, Impella RP, and Impella CP in their proper positions.

#### Managing Complications: Hemolysis, Thrombosis, and Vascular Issues

Impella-related complications are heterogeneous; their approximate incidences, together with key considerations for early detection and prevention/mitigation, are summarized in Table 2, along with a concise, practical bedside management algorithm.

**Table 2:** Complications associated with Impella device therapy and related preventive strategies and management

Complication	Approximate incidence	Main mechanisms	Preventive strategies	Management
Hemolysis	20%–30% (up to 40% in prolonged support)	Shear stress, suction events, low preload, right ventricular heart failure, thrombosis, malposition	Position device correctly (SmartAssist guidance), maintain adequate preload, avoid suction, ensure sufficient purge flow	Check pfHb, LDH, haptoglobin daily; conduct transthoracic echocardiograpy every 8 hours to assess device position; optimize volume status; consider inotropes, RV support or repositioning or switch to 5.5 device; if severe, consider iNO, plasmapheresis, or hemadsorption
Pump thrombosis	5%–10%	Low flow, inherent prothrombotic states, purge obstruction, prolonged use	Maintain UFH (aPTT 40–80 seconds; anti-Xa 0.5-0.7 IU/mL), monitor purge pressure (<600 mmHg), prevent stasis	Increase anticoagulation, replace device if persistent; consider thrombectomy or tPA in selected cases
Vascular complications	10%–20% overall	Large sheath size, calcified or small vessels, improper puncture site	Preprocedural CT or US imaging, ultrasound-guided puncture, preclosure devices, SHiP access	Endovascular repair or covered stent; manual compression or thrombin injection for pseudoaneurysm
Acute limb ischemia	Up to 12%	Arterial obstruction, spasm, embolization	Use smallest feasible sheath, assess peripheral anatomy, frequent distal pulse checks	Urgent US/angiography; thrombus aspiration, thrombectomy, or vascular surgery
Bleeding/Hematoma (including retroperitoneal)	5%–15%	High puncture site, anticoagulation, prolonged support	Ultrasound-guided puncture, preclosure, maintain therapeutic ACT/aPTT range	Balloon tamponade, covered stent; surgical repair if persistent

*Notes*. Complication management algorithm (at bedside): detect (labs/alarms/clinical) → classify (device vs. access vs. systemic) → verify position and purge → optimize preload/anticoagulation → RPM trial (if safe) → targeted fix (reposition, exchange, vascular intervention) → escalate/de-escalate device (e.g., upgrade to 5.5 for required flow at lower RPM, or remove when criteria met).

Abbreviations: ACT, activated clotting time; aPTT, activated partial thromboplastin time; CT, computed tomography; iNO, inhaled nitrous oxide; LDH, lactate dehydrogenase; pfHb, plasma-free hemoglobin; RPM, revolutions per minutes; RV, right ventricle; SHiP, Single access for High-risk PCI; tPA, tissue plasminogen activator; UFH, unfractionated heparin; US, ultrasound.

#### Hemolysis

Hemolysis occurs in at least one-quarter of Impella-supported patients due to shear stress, flow acceleration, and direct contact of red blood cells with the device.^93^ This leads to red blood cell stretching and rupture and plasma-free hemoglobin (pfHb) release,^94^, ^95^, ^96^ triggering thrombosis, vasoconstriction, and potential organ failure, particularly in the kidneys.^97^^,^^98^ Overinsertion, hypovolemia, or right HF can cause suction events,^99^^,^^100^ while low or obstructed flush flow due to clot adhesion further contributes to hemolysis. Clinically, “tea-colored urine” and jaundice should be confirmed by elevated pfHb, lactate dehydrogenase (LDH), phosphate, bilirubin, and potassium with decreased hemoglobin and haptoglobin.^101^ There is no standardized threshold, but pfHb levels >20 mg/dL suggest hemolysis.^102^ Alternatively, hemolysis may be suspected if there is a decrease in hemoglobin, a need for transfusion in addition to a decrease in haptoglobin, or an increase in LDH.^103^ Daily blood sampling should be encouraged for early detection.

The SmartAssist technology provides positioning data using an optical aortic sensor. Negative systolic/diastolic pressures indicate malposition and require downgrading to P2 prior to echocardiographic repositioning.^83^

On the other hand, negative diastolic but normal systolic ventricular curves, hypovolemia (low pulmonary artery wedge pressure [PAWP]) or right HF (low/normal PAWP, high right atrial pressure [RAP], PA pulsatility index <1) should be differentiated by right heart catheterization.^82^ Management includes fluids or inotropes/additional RV support, respectively.^83^ Thrombosis should be suspected in hemolysis, especially with high purge pressures or previous coagulopathy, requiring device replacement.^104^ Rare causes including sickle cell anemia or thrombotic microangiopathy should be considered if other causes were excluded. In severe cases, inhaled nitrous oxide, plasmapheresis, or hemadsorption can counteract pfHb-induced vasoconstriction.^104^^,^^105^ Notably, hemolysis does not invariably improve with revolutions per minutes reduction; in selected patients, upsizing to Impella 5.5 can deliver the required flow at lower rotational speed and may mitigate shear-related hemolysis.

#### Thrombosis

Thromboembolic complications have been documented in approximately 10% of Impella implantations in international registries.^80^^,^^106^ The device features a unique purge system that continuously infuses a dextrose-based heparin solution through the microcavities adjacent to the pump bearings.^104^ This design minimizes blood ingress and clot formation within the device. However, prolonged use, low-flow conditions, and inherent prothrombotic states may precipitate acute Impella thrombosis, thereby increasing the risk of embolizations and device failure, which necessitates adjunctive systemic anticoagulation.^107^ Clinically, patients may present with worsening shock, significant hemolysis, AKI, limb ischemia, or cerebrovascular events. A progressive rise in purge pressure above 600 mmHg, coupled with a low flushing rate (<2 mL/h) and elevated LDH and D-dimer levels, are indicative of thrombosis.^108^ Echocardiography or contrast-enhanced computed tomography (CT) could support the diagnosis.^109^ Management typically involves initiating unfractionated heparin at 12 UI/kg with serial monitoring of activated partial thromboplastin time (aPTT) (target: 40–80 seconds) and anti-Xa levels (target: 0.5–0.7 IU/mL) every 4 to 6 hours.^104^ Discrepancies between these measurements may prompt evaluation of antithrombin levels or consideration of alternative anticoagulants (e.g., biluvardin).^104^ Despite optimized anticoagulation, persistent thrombosis, or device dysfunction may require removal of the Impella or transition to other MCS modalities (e.g., ECMO).^110^^,^^111^ Several case series supported alternative approaches, including mechanical thrombectomy or tissue plasminogen administration.^108^^,^^109^

#### Vascular Complications

Despite heterogeneous definitions in previous studies,^9^^,^^106^^,^^112^, ^113^, ^114^ vascular complications are the most common adverse events in large-bore sheath procedures, impacting procedural success, patients’ prognosis, and hospital stay.^115^ Preprocedural imaging such as CT angiography or digital subtraction imaging helps to assess iliofemoral anatomy and guide optimal access site selection.^116^^,^^117^ The common femoral artery is the preferred access site due to its size and compressibility,^118^ except in those with concomitant PAD, concentric vessel calcification, tortuosity, obesity, or small vessels.^119^ In these patients, transaxillary and caval-aortic techniques are alternative approaches. An ultrasound-guided single anterior puncture with the smallest access needle feasible ensures precise cannulation and avoids calcified areas.^117^^,^^118^ The use of Single access for High-risk PCI (SHiP) can further reduce this to one versus multiple sterile fields.^120^ Preclosure techniques using ProGlide or ProStar devices facilitate hemostasis.^121^^,^^122^ MANTA or AngioSeal systems are viable alternatives when preclosure is unfeasible.^123^

Retroperitoneal hematoma is feared in those with high arteriotomy access, prolonged support, and supratherapeutic anticoagulation.^124^ Balloon inflation and subsequent covered stent deployment ensure coverage of the perforation site. Conversely, pseudoaneurysms are associated with arteriotomies below the common femoral artery bifurcation or multiple arterial punctures.^118^ In most cases, either ultrasound-guided manual compression or thrombin injection is effective management strategy.^118^

**Figure 5:**
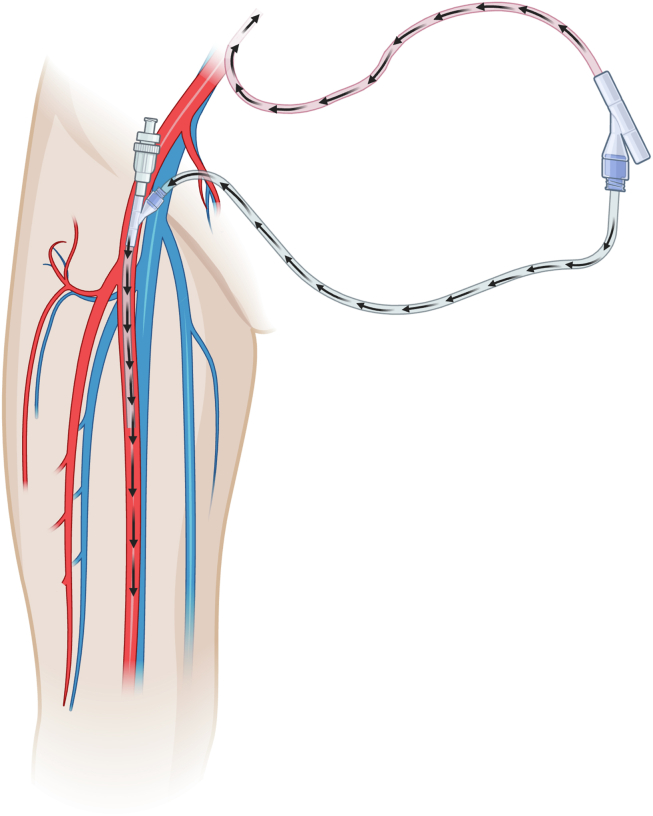
Reperfusion cannula.

Acute limb ischemia, which occurs in up to 12% of patients, requires immediate assessment with ultrasound or angiography.^2^ Treatment options include thrombus aspiration, thrombectomy or, in severe cases, vascular surgery. A reperfusion cannula can be added to reduce the risk of limb ischemia (see Figure 5).

In conclusion, device size and access sheath diameter are key determinants of complication rates. Larger-bore systems (e.g., Impella 5.0/5.5) are associated with higher vascular complication risk^61^ but may reduce hemolysis due to lower shear stress. Of note, decreasing pump speed alone does not always mitigate hemolysis; in selected cases, escalation to the 5.5 device can improve hemocompatibility and unloading efficiency.^125^

### Integrating Impella Data Into Overall Patient Management

Management of Impella-supported patients requires continuous integration of hemodynamic, laboratory, and imaging data to ensure optimal outcomes.^86^

Echocardiography and SmartAssist technology data should be evaluated daily to interpret an early device malposition or significant flow changes.^87^ However, PAC complements SmartAssist information to optimize pharmacological therapy (fluids or inotropes), differentiate hypovolemia from advanced CS, assess the right circulation (RV output; central venous pressure; PA pulsatility index), and guide weaning.^126^ Daily serum lactate, mixed venous oxygen saturation, and urine output monitor for subclinical organs dysfunction.^86^ During the first days after Impella implantation, monitoring of LDH, pfHb, and bilirubin is essential to prevent hemolysis and adjust flow or positioning as needed. Thrombosis prophylaxis relies on anticoagulation markers (partial thromboplastin time [PTT], international normalized ratio [INR], anti-Xa).^86^ The cannulated limb must be carefully assessed, as antegrade perfusion is not used. Therefore, skin color, arterial Doppler, and near-infrared spectroscopy can be used to detect potential limb ischemia.^118^

## Clinical Applications

### Cardiogenic Shock: Evidence From Trials and Real-World Data

MCS is pivotal for managing CS, with a substantial body of recent literature addressing its role. CS is a clinical syndrome characterized by severe impairment in cardiac function, reduced CO, and inadequate organ perfusion, necessitating pharmacological or MCS.^33^ Distinctions in pathophysiology exist between CS secondary to ADHF-CS and following AMI-CS. ADHF-CS typically presents with lower lactate levels, more frequent right ventricular dysfunction, and a higher incidence of hepatorenal organ damage due to chronically reduced CO, often requiring vasodilators and ventricular unloading.^127^ In these cases, IAoBP is supposed to improve organ perfusion.^4^^,^^128^ However, in a recently published randomized controlled trial, routine early IAoBP plus standard care, compared with standard care, did not significantly improve survival or successful bridging to heart replacement therapies in patients with ADHF-CS.^129^

In contrast, AMI-CS generally involves smaller LVs and an acute decrease in CO, necessitating support devices capable of actively generating forward flow.^130^ Despite the use of MCS, short-term mortality for CS remains elevated (20%–60%), and no pharmacological therapy has demonstrated a definite survival advantage. ^80^^,^^131^

In recent years, the Impella device has gained traction in AMI-CS for its ability to unload the LV by reducing EDP, increasing CO, lowering myocardial oxygen demand, and enhancing coronary perfusion.^93^ Before 2024, safety and efficacy data for mAFP in CS—largely derived from observational studies—were controversial,^3^^,^^59^^,^^80^^,^^131^, ^132^, ^133^ prompting only weak recommendations in the 2023 European guidelines on acute coronary syndromes.^134^ In many of those studies, Impella 2.5 was placed post-PCI, often during an early learning curve, which may explain the lack of observed benefit. However, several analyses indicate improved outcomes when Impella is implanted before PCI, as highlighted by a meta-analysis on the topic^135^ and other studies.^13^^,^^136^ The ST-segment Elevation Myocardial Infarction (STEMI) Door-to-Unload (DTU) pivotal trial is currently assessing whether pre-PCI Impella placement reduces infarct size and improves prognosis.^137^

Multiple observational studies comparing Impella with IAoBP have not shown a mortality benefit and instead reported higher complication rates with Impella (bleeding, hemolysis, vascular injury),^136^^,^^138^^,^^139^ supported by propensity-matched analyses.^14^^,^^15^^,^^140^ The first randomized trial demonstrating both the feasibility and safety of Impella in CS—while showing greater hemodynamic improvement but no mortality difference versus IAoBP—was Impella LP 2.5 versus IABP in Cardiogenic Shock (ISAR-SHOCK) trial (2008).^141^ The subsequent IMPella versus IABP Reduces mortality in STEMI patients treated with primary PCI in Severe cardiogenic SHOCK (IMPRESS) trial failed to show an acute and 5-year mortality advantage in AMI-CS and mechanically ventilated patients receiving Impella, primarily because of high complication rates.^3^^,^^142^

The recently published DANGER-SHOCK trial is the first to demonstrate that adding Impella to standard care in 355 STEMI patients with CS reduced 6-month mortality, albeit with an increased risk of severe bleeding, limb ischemia, hemolysis, device failure, and worsening aortic regurgitation.^11^ Subanalyses revealed that Impella use decreases vasopressor/inotrope requirements, maintains hemodynamic stability, and accelerates lactate clearance.^16^ Shock severity, assignment to mAFP, and device-related complications predicted AKI, which was associated with higher mortality. Nevertheless, Impella use yielded lower 180-day mortality regardless of AKI.^143^ This benefit was attenuated in older patients, as seen in a recent substudy.^144^ In DANGER-SHOCK, approximately 20% of patients had undergone resuscitation after cardiac arrest, though only 2% had brain injury. Prior resuscitation is a known predictor of worse prognosis in mAFP-treated patients^132^^,^^145^—likely due to irreversible brain damage—yet Impella may still benefit certain individuals recovering from cardiac arrest.^146^ Importantly, DANGER-SHOCK’s generalizability is constrained by trial features: inclusion was limited to infarct-related CS, implantation timing and device management occurred in experienced centers, the trial was open-label with higher bleeding and vascular events in the device arm, and crossover/protocol deviations may have influenced effect estimates. These factors warrant cautious extrapolation beyond the studied phenotype. Following the positive results of Danish-German Cardiogenic Shock trial (DanGer Shock), the RECOVER IV randomized trial (NCT05506449), which was designed to compare early versus standard Impella CP implantation in AMI-CS, was suspended after its independent Data and Safety Monitoring Board judged that clinical equipoise was no longer present and recommended termination, and enrollment was halted. Consequently, the 2025 American guidelines on acute coronary syndromes upgraded mAFP to a Class IIa (Level of Evidence B) recommendation.^147^ Further research is necessary to confirm mAFP benefits in broader CS populations.^5^

**Figure 6:**
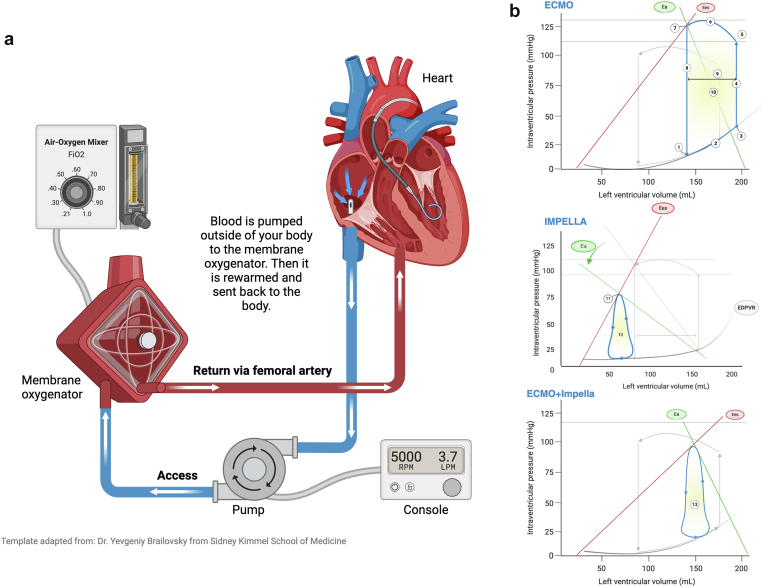
Schematic representation of ECMO and Impella support and effects on left ventricular PV loops. (a) Venoarterial ECMO drains venous blood through a femoral cannula, pumps it through a membrane oxygenator, and returns it to the systemic circulation via the femoral artery. An Impella device is positioned across the aortic valve into the LV, providing active unloading. (b) Hemodynamics effects on left-ventricle PV loops: ECMO increases left ventricular afterload and may elevate end-diastolic pressure and volume. The Impella, by actively unloading the ventricle, reduces end-diastolic pressure and volume with a triangular PV loop due to loss of isovolumic phases. Combined ECMO + Impella mitigates ECMO-induced afterload increase while providing ventricular unloading, resulting in improved LV mechanics. (1) Mitral valve opens. Ventricular fast filling starts. (2) Diastole progresses to ventricular slow filling. (3) Mitral valve closes. Ventricular diastole ends. (4) Isovolumetric contraction period. (5) End-diastolic pressure. Aortic valve opens. (6) Ejection period. (7) Ventricular systole ends. Aortic valve closes. (8) Isovolumetric relaxation period. (9) Stroke volume. (10) Cardiac work. (11) Loss of isovolumetric contraction, end-diastolic pressure equals end-systolic pressure in a triangular shape PV-loop. (12) Cardiac work during Impella support. (13) Cardiac work during ECMELLA support. Abbreviations: Ea, arterial load; ECMELLA, venoarterial extracorporeal membrane oxygenation plus Impella support; ECMO, extracorporeal membran oxygenation; EDPVR, end-diastolic pressure-volume relationship; LV, left ventricle; PV, pressure-volume; Ees, end-systolic elastance.

Another potential indication for Impella in AMI-CS is in patients requiring extracorporeal life support (ECLS), which diverts blood from the venous system, oxygenates it, and returns it to the arterial circulation. The retrograde flow of VA-ECMO provides adequate perfusion but significantly elevates the LV afterload, a disadvantage in CS. This can lead to LV distension, rising LV EDP, pulmonary congestion, impaired aortic valve opening, and reduced coronary perfusion. An LV-unloading device such as Impella can vent the LV and lower filling pressures. In certain scenarios (e.g., refractory arrhythmias, prolonged cardiopulmonary resuscitation [CPR], severe biventricular failure), VA-ECMO may be the only MCS option. In such cases, combining VA-ECMO with a device that unloads the LV, such as Impella, may be considered (see Figure 6). The Extracorporeal Life Support in Infarct-Related Cardiogenic Shock (ECLS-SHOCK) trial showed no short-term (30 days) or long-term (1 year) survival benefit from routine ECLS in AMI-CS, particularly among patients with advanced shock stages (SCAI D-E) and limited LV unloading (<6%).^6^^,^^148^ Retrospective analyses and meta-analyses have suggested reduced mortality with ECMELLA (VA-ECMO plus Impella support) compared to VA-ECMO alone.^7^^,^^8^^,^^149^, ^150^, ^151^ ECMELLA may also aid VA-ECMO weaning by allowing early ECMO removal while maintaining LV support.^152^ However, these data are observational and subject to selection and treatment-timing confounding. No randomized trial has yet evaluated LV unloading in VA-ECMO for CS, but the ongoing Early Left Ventricular Unloading After Extracorporeal Membrane Oxygenation (EARLY-UNLOAD) (NCT03431467) and Left Ventricular Unloading to Improve Outcome in Cardiogenic Shock Patients on VA-ECMO (UNLOAD-ECMO) (NCT05577195) trials aim to address this gap.

### High-Risk PCI: Safety and Effectiveness

High-risk PCI (Complex High-risk Indicated PCI [CHIP,]) represents a subset of procedures performed in patients with complex clinical and anatomical characteristics. CHIP generally refers to PCI performed in patients with both high clinical risk (e.g., severe LV dysfunction, advanced age, renal insufficiency, diabetes) and high anatomical complexity (e.g., multivessel coronary artery disease, left main, severe calcification, bifurcations, or chronic total occlusions). However, a universal definition is lacking, and different studies have adopted heterogeneous inclusion criteria. Only older PCI guidelines^153^ tried to give a definition based exclusively on myocardium at risk and cardiac function. Only recently did some authors attempt to define CHIP by identifying predictors of mortality to estimate the mortality risk with a clinical score.^154^, ^155^, ^156^

The goal of CHIP procedures is to provide revascularization to patients who may otherwise be deemed unsuitable for surgery and thereby improve both their quality of life and prognosis.^157^ In these patients, transient hemodynamic compromise is anticipated, particularly by anatomical and cardiac function parameters; MCS devices, and especially Impella, are increasingly employed to stabilize hemodynamics during these interventions, providing circulatory assistance, reducing LV workload, and maintaining adequate organ perfusion.^158^^,^^159^ In a substudy of the PROTECT III trial, patients who experienced LOP during CHIP supported by Impella had higher rates of major adverse cardiovascular events (MACE), cerebrovascular events, AKI, and death within 90 days; worse hemodynamic parameters, more than comorbidities and anatomical complexity, defined patients with LOP.^160^ Patients who hypothetically experienced LOP without Impella support may have undergone more severe hemodynamic deterioration and, because of procedural complications, complete revascularization could not have been pursued. However, there is still debate whether this kind of procedure should be performed with MCS because of conflicting data coming especially from observational studies.

The use of Impella in CHIP procedures has been associated with improved procedural outcomes and enhanced safety profiles. A recently published meta-analysis evaluating rates of events in Impella-CHIP found acceptable complication incidence: in-hospital mortality 5%; vascular complications and bleeding 2.5% and 6%, respectively; and postprocedural dialysis 4%.^161^ The results align with other large registries, except for higher bleeding complication rates (up to 13%).^162^ The PROTECT II trial is the only randomized controlled trial comparing Impella and IAoBP in CHIP.^9^ The trial did not meet its primary endpoint of 30-day major adverse events. However, secondary analyses and longer-term follow-up suggested potential benefits with Impella, including reduced repeat revascularization and improved outcomes in selected patients. The PROTECT II-Impella cohort is a landmark population used in the years after by observational studies as a control, both comparing nonprotected CHIP and more recent Impella-protected procedures.^163^ The PROTECT III trial is a single-arm study evaluating contemporary Impella-supported procedures compared to the historical cohort; a significative improvement in major adverse cardiovascular and cerebrovascular events (MACCE), repeat revascularization, hypotension during support, and need for cardiopulmonary resuscitation or ventricular arrhythmia rates were observed within the more recent population, both for in-hospital and 90-day follow-up.^148^ Moreover, bleedings were less frequent in the PROTECT-III cohort (1.8% from 9.3%). Despite these reassuring results, routine protected PCI is still far from being recommended because of conflicting data coming from observational studies, highlighting no differences or worse harmful use of MCS-supported PCI compared to unprotected procedures.^164^^,^^165^ These findings highlight the ongoing difficulty in identifying the right patient to undergo procedures with circulatory support. In this context, patients with unfavorable baseline hemodynamics, large areas of myocardium at risk, and reduced ejection fraction could represent the ideal target. Pursuing complete revascularization, as demonstrated by other trials,^166^ may improve long-term outcomes, and it can be facilitated with the use of devices such as Impella in these patients.^167^, ^168^, ^169^ Overall, randomized data supporting routine use of Impella in CHIP remain limited, with PROTECT II being neutral for its primary outcome. The majority of evidence comes from registries and observational studies, which suggest improved safety and efficacy but also report conflicting results. Thus, while protected PCI may facilitate complete revascularization and improve outcomes in selected high-risk patients, definitive conclusions require further randomized trials.

### Impella vs. Other Devices

Compared to other MCS devices, such as IAoBP and VA-ECMO, Impella offers distinct advantages in CHIP procedures.

Unlike IAoBPs, which rely on diastolic augmentation, Impella actively unloads the LV, reducing myocardial oxygen demand and improving coronary perfusion. The only ranodmized clinical trial (RCT) comparing the 2 devices is the PROTECT II trial, which showed overlapping safety profiles with an important trend for reduced MACE rate at 90 days and lower hypotensive events in 3-vessel disease.^9^ Large subsequent registries offered conflicting results: from the same large database, a nonmatched comparison showed higher rates of death and bleeding in the Impella group, while a propensity-adjusted analysis demonstrated lower rates of mortality, myocardial infarction, and CS with Impella compared to IaoBP.^170^^,^^171^

In contrast to ECMO, which provides full cardiopulmonary support, Impella is less invasive, easier to deploy, and associated with lower complication rates in the CHIP population. There is a paucity of data directly comparing Impella and ECMO in nonemergent CHIP because of intrinsic differences in timing and support of the 2 devices in different settings: all the observational studies results are consistent in affirming a higher mortality, bleeding, and complications rate with ECMO compared to Impella. While ECMO may be more appropriate for patients in CS or severe respiratory failure, Impella is specifically tailored to address the hemodynamic challenges of high-risk PCI, making it a highly suitable choice in these patients.^162^^,^^172^^,^^173^

In conclusion, Impella as mechanical support in high-risk PCI is backed by evidence that is not always consistent but shows a favorable trend for better short- and long-term outcomes compared to unprotected procedures or those performed with the assistance of other devices. Patient selection is crucial, and the most appropriate criteria likely remain to be defined through dedicated studies. Nonetheless, with the aim of improving survival and quality of life, circulatory support provides a safety net for the operator by enabling the pursuit of complete revascularization in patients otherwise ineligible for cardiac surgery.

### Right Ventricular Failure: Unique Applications of the Impella RP

For the underlying physiology of Impella support in right ventricular failure, refer to “Physiology of Hemodynamic Support: Differences in Physiology for Left- vs. Right-Sided Support” earlier in the article.

The treatment of right ventricular failure should focus on addressing the underlying cause with the immediate goal of maintaining adequate perfusion pressure, optimizing preload, and reducing afterload to enhance RV contractility and improve CO.^174^

When optimal medical therapy fails or is insufficient to maintain hemodynamic stability,^52^ MCS may be the most beneficial option.^53^^,^^175^ MCS serves as a temporary measure until either RV function recovers or advanced HF treatments are pursued. Temporary MCS devices help unload the RV and promote recovery, making them crucial in clinical practice, as the RV is known to be more resilient than the LV following injury.^175^ Given that severe RV failure is often more reversible compared to LV failure, the use of temporary percutaneous support devices may facilitate faster recovery.

Several MCS options that are frequently used for RV failure vary in the level and duration of support provided. In recent years, the development of percutaneous MCS changed the landscape of RV failure treatment, providing less invasive alternatives to surgical device.^48^ One such option is the Impella RP catheter, a minimally invasive, percutaneously implanted microaxial pump that acts as a direct RV bypass.^176^ The Impella RP is commonly used following LVAD implantation, postcardiotomy, or after myocardial infarction^177^ or acute pulmonary embolism in cases of acute RV failure.^178^, ^179^, ^180^ It has been approved for up to 14 days of support.^181^

The Impella RP provides circulatory support by increasing RV CO while simultaneously reducing central venous pressure,^50^ ultimately restoring systemic perfusion.^182^ This allows for the gradual weaning from inotropic and vasopressor support,^49^ either bridging the patient to recovery or to long-term MCS.^183^ Notably, in case of isolated RV failure with preserved LV function, the device does not impact LV afterload but improves preload delivery to the LV.^49^

The efficacy of the Impella RP was first evaluated in 2015 in the RECOVER RIGHT study, a prospective, multicenter, single-arm outcomes trial involving 30 patients (12 with AMI and postcardiotomy, 18 following LVAD implantation). The study demonstrated improved survival and reduced morbidity at 30 days in patients with RV failure refractory to medical treatment.^47^ In 2018, similar findings were reported by Anderson et al. in a prospective cohort study of 60 patients with refractory RV failure following LVAD implantation,^184^^,^^185^ postcardiotomy,^186^ heart transplant, or AMI.^187^

Overall, current data suggest that, in offering the potential for rapid patient stabilization, the Impella RP is a valuable therapeutic option for managing patients suffering from CS secondary to RV failure. However, technical and management challenges remain due to the limited scientific data available. Despite these challenges, the device represents a promising bridge from therapy to recovery or definitive treatment.

## Controversies and Limitations

### Cost-Effectiveness and Financial Burden

The clinical uptake of Impella in both CS and high-risk PCI has prompted debate regarding its cost compared with alternatives such as the IAoBP and VA-ECMO.

Economic evaluations of the Impella have been addressed through retrospective analyses and cost-effectiveness models, with most studies adopting a healthcare system (payer) perspective, examining index hospitalization costs and, in some cases, extending the analysis to lifetime projections.

Although Impella has higher acquisition costs and potential complications, these are often offset by improved survival, shorter intensive care unit and hospital stays, and enhanced recovery.

Multiple analyses suggest that in appropriately selected patients, Impella yields favorable outcomes (Table 3):

• Cost Savings (United States data): Total episode-of-care cost savings have ranged from $45,000 to $55,000 per patient when compared with ECMO or surgical alternatives. For instance, a pivotal study demonstrated a $45,000 cost savings alongside a 58% reduction in mortality for pVADs in CS due to coronary atherosclerosis.^89^ Similarly, a retrospective claims analysis comparing pVADs and ECMO observed $54,571 in episode-of-care savings with pVAD use, largely attributed to fewer complications.^91^
• Incremental Cost-Effectiveness Ratios: Retrospective cost-effectiveness evaluations in both the United States and Europe corroborate these findings. Roos et al., for example, reported an incremental cost-effectiveness ratio of approximately €26,000 per quality-adjusted life year for Impella versus IAoBP in high-risk PCI,^92^ a value well below conventional willingness-to-pay thresholds.
• Wider Economic Impact: Beyond immediate hospitalization, Impella’s capacity to facilitate native heart recovery may avert the need for more advanced and costly treatments such as heart transplantation or durable LVADs. Reflecting such data, the United Kingdom’s National Institute for Health and Care Excellence has acknowledged the device’s utility in select high-risk PCI populations.

**Table 3:** Summary of key economic analyses of Impella support

Study	Clinical setting	Cohort size	Comparator	Perspective	Main assumptions	Key findings
Stretch et al., 2014^89^	National inpatient database (cardiogenic shock)	>20,000	Conventional support	United States payer	Reduced length of stay and mortality offset device cost	Cost savings ≈ $45,000 per patient
Maini et al., 2014^90^	Emergent support	637	Surgical LVAD	Hospital	Shorter stay, improved survival	Lower total cost and mortality with Impella
Vetrovec et al., 2020^91^	AMI-related cardiogenic shock	>10,000	VA-ECMO	United States payer	Fewer complications and shorter ICU stay	Episode-of-care savings ≈ $54,500
Roos et al., 2013^92^	High-risk PCI	250	IAoBP	European payer	Modeled survival gain and reduced rehospitalization	ICER ≈ €26,000 per QALY (favorable)

Abbreviations: AMI, acute myocardial infarction; IAoBP, intra-aortic balloon pump; ICER, incremental cost-effectiveness ratio; ICU, intensive care unit; LVAD, left-ventricular assist device; PCI, percutaneous coronary intervention; QALY, quality-adjusted life year; VA-ECMO, venoarterial extracorporeal membrane oxygenation.

Nonetheless, economic benefits are highly dependent on patient selection, operator experience, and institutional procedural volume.^9^ Centers with limited exposure or suboptimal use may experience attenuated economic value. Furthermore, reimbursement for microaxial pumps varies widely across health systems, and in settings with comparatively high reimbursement, economic incentives may unintentionally influence device use, underscoring the need for transparent, protocol-driven indications.

Consequently, large-scale, prospective, multicenter cost-utility analyses—incorporating postdischarge resource utilization, long-term quality-of-life outcomes, and diverse healthcare systems—remain essential to fully validate Impella’s financial viability across clinical settings.^140^

### Evidence Gaps and Trial Limitations

Although enthusiasm for Impella is increasing, existing data are heterogeneous. Much of the evidence derives from observational registries or single-center studies with limited sample sizes.^14^^,^^15^ Early randomized trials, such as ISAR-SHOCK and IMPRESS, demonstrated hemodynamic improvements but did not conclusively establish mortality benefits over IAoBP in small, specialized populations.^3^^,^^141^

In a recent level meta-analysis including CS patients supported with Impella devices (both percutaneous and surgical), pooled short-term mortality (defined as 30-day mortality or in-hospital mortality) was 46.5%, with no significant differences in subgroups (including CS etiology). Pooled long-term mortality was 41.8%, 51.1%, and 54.3% at 90 days, 6 months, and 1 year, respectively.^188^

More recently, the DANGER-SHOCK trial provided randomized evidence suggesting mortality reductions in infarct-related CS.^11^ Nevertheless, questions linger about the generalizability of these findings to other shock subtypes or complex comorbidities. Additionally, outcome variability likely stems from differences in institutional protocols and varying operator proficiency.^9^

### Ethical Considerations for End-Stage or Marginal-Benefit Cases

Ethical dilemmas arise when short-term mechanical support is considered in patients with end-stage cardiac disease or those who lack candidacy for advanced therapies such as transplantation. Although Impella can stabilize hemodynamics acutely, complications—ranging from hemolysis to bleeding and infection—can prolong suffering without ensuring a clear survival or quality-of-life benefit.^10^

In cases ineligible for definitive interventions, extended use of pVADs may result in a “bridge to nowhere,” raising moral concerns about resource utilization. Early palliative care input and transparent discussions with patients and families are pivotal in delineating goals of care.^33^ By aligning treatment decisions with clinical realities and patient values, clinicians can mitigate the risks of overburdening therapy in this vulnerable population.^66^

## Future Perspectives

### Innovations in Device Technology: Miniaturization, Monitoring, and Thrombogenicity

Continuous technological refinements are central to advancing the Impella platform. A primary focus is miniaturization, exemplified by the Impella ECP, which targets a 9 Fr expandable sheath without compromising flow.^65^ Reduced sheath sizes could lower vascular complication rates, expedite device deployment, and improve feasibility in patients with PAD.

Parallel efforts to enhance real-time monitoring are also underway. SmartAssist, available in specific Impella models, uses optical sensors to detect device malposition or suction events promptly, potentially reducing hemolysis.^63^ Future versions may integrate artificial intelligence to automatically modulate pump speed based on instantaneous hemodynamic data, minimizing human error.

The latest device of the family, Impella Flex RP with SmartAssist, may offer benefits such as improved patient mobility during support (by enabling internal jugular vein insertion) and an easier cannulation pathway. Though clinical experience with this device remains limited, initial reports suggest successful support and weaning in early patient cohorts. As with the standard Impella RP, the “Flex” version can be used in conjunction with a left-sided Impella for full biventricular assistance (BiPella). Further data and larger studies are warranted to clarify its safety and efficacy relative to earlier Impella RP models.

Another area of active research is the reduction of thrombogenicity. Innovations in impeller coatings and rotor designs aim to minimize shear forces and thereby curb platelet activation and thrombus formation. Enhanced purge systems and sensor-driven purge pressure algorithms are also being explored to further reduce clotting risk while maintaining adequate lubrication of pump bearings. If validated in clinical studies, such modifications could significantly diminish device-related thromboembolic complications.

### Integration With Hybrid Support Strategies

**Figure 7:**
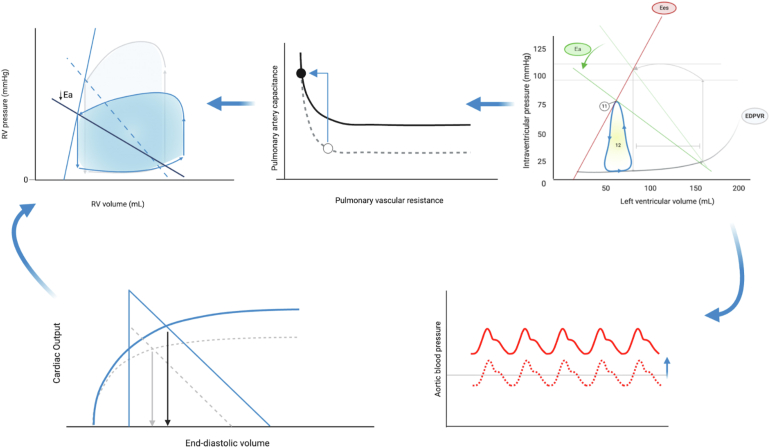
Schematic representation of right ventricular hemodynamic effects of left-sided Impella support. By lowering left ventricular end-diastolic volume/pressure, the pressure-volume loop becomes triangular with a reduction in pulmonary artery wedge pressure and an increase in pulmonary artery capacitance. Due to the hyperbolic curve, a small reduction in pulmonary vascular resistance leads to a significant increase in pulmonary artery capacitance. This reduces right ventricular afterload (↓Ea) and, together with enhanced systemic venous return, augments right ventricular output. The balanced interaction preserves left ventricular preload while increasing systemic arterial pressure, with pulsatile flow maintained as long as the native left ventricular stroke work is preserved. Abbreviations: Ea, arterial load; EDPVR, end-diastolic pressure-volume relationship; Ees, end-systolic elastance.

ECMELLA has emerged as a novel approach for refractory CS.^7^^,^^8^ While ECMO confers potent circulatory and oxygenation support, it also increases left ventricular afterload. Impella offsets this limitation by unloading the ventricle, potentially enhancing coronary perfusion and limiting pulmonary congestion (see Figure 7). Retrospective analyses have yielded encouraging short-term results, although dual large-bore cannulation, anticoagulation needs, and bleeding risks remain substantial.^104^

Recent findings from the UNLOAD-ECMO investigation suggest that early LV decompression during ECMO support can be associated with improved outcomes in carefully selected patients. Although large prospective trials are still needed to standardize patient selection, timing of implantation, and weaning protocols, these emerging data point toward ECMELLA as a potentially established treatment option for the most severe shock phenotypes.^189^

### Prospects for New Indications and Ongoing Trials

Research continues to expand Impella’s horizons beyond acute CS and high-risk PCI. The STEMI-DTU trial (NCT03947619) investigates whether LV unloading prior to reperfusion can reduce infarct size and improve long-term outcomes in STEMI.^142^ Similarly, the IMpella-Protected cArdiaC Surgery Trial (IMPACT, NCT05529654) is evaluating prophylactic Impella use in high-risk surgical patients to prevent or mitigate postcardiotomy shock.

Additional targets include refractory ventricular tachycardia ablation and BiPella in severe biventricular failure. A recent multicenter retrospective analysis of 20 patients with BiPella support reported a 50% in-hospital mortality rate but also found that younger age, fewer vasopressors, and lower right ventricular afterload were associated with better outcomes.^64^ These investigations leverage the range of Impella devices—spanning 2.5 to 5.5 L/min capacity—to customize support. Furthermore, real-world registries continue to refine patient selection and procedural best practices, guiding future design and clinical protocols.

## Conclusions

In light of the evidence reviewed, conclusions are summarized below into what is established, what remains uncertain, and future research priorities.

### What is Established

• Impella provides active ventricular unloading that can stabilize hemodynamics and support myocardial recovery when viable myocardium remains and injury is potentially reversible.
• In infarct-related CS, randomized and contemporary data suggest a possible survival benefit with judicious, protocolized use, balanced by higher rates of bleeding and vascular complications. However, pending confirmatory evidence, any expansion of microaxial support should be selective and protocol-driven given trial limitations and variability in practice.
• In high-risk PCI, Impella’s principal value is procedural in facilitating hemodynamic stability and completeness of revascularization. A mortality benefit has not been demonstrated.

### What Remains Uncertain

• Generalizability beyond infarct-related shock (e.g., myocarditis, postcardiotomy, decompensated chronic HF) and the optimal timing of implantation across phenotypes.
• Net clinical benefit versus alternatives (IAoBP, VA-ECMO, or hybrid approaches) in different risk profiles, and how program experience/reimbursement structures influence outcomes and equity of access.
• Best practices to minimize complications (hemolysis, vascular injury, thrombosis), including standardized anticoagulation, access strategy, and weaning protocols.
• Economic value across health systems, recognizing heterogeneous reimbursement and ethical considerations (e.g., “bridge to nowhere” scenarios).

### Future Research Priorities

• Confirmatory trials to define who benefits most and under what conditions, with prespecified subgroup analyses and standardized care pathways.
• Timing and selection studies (e.g., pre- vs. post-revascularization strategies; integration with SCAI staging, lactate, MAP, and frailty).
• Comparative and strategy trials of Impella vs. IAoBP/VA-ECMO and hybrid strategies (e.g., ECMELLA), powered for mortality and complications.
• Complication-reduction programs (e.g., scalable bundles for access planning, anticoagulation, hemolysis prevention, and structured weaning) and evaluation of device refinements (e.g., lower-thrombogenicity components, miniaturized sheaths, and smart or artificial intelligence–assisted control systems).
• Robust health-economic analyses across jurisdictions, capturing postdischarge resource use and patient-reported outcomes.

## Ethics Statement

This article is a narrative review of previously published studies and does not report new experiments involving human participants, human data, human tissue, or animals. Therefore, institutional review board approval and informed consent were not required. The authors confirm that the work was conducted in accordance with relevant ethical guidelines for scientific publication.

## Funding

The open-access publication fees for this review were funded by 10.13039/100020297Abiomed, Inc. (22 Cherry Hill Drive, Danvers, MA 01923, USA). The company had no role in the conception, design, or intellectual content of the manuscript, and it did not influence the drafting, revision, or final approval of the work.

## Disclosure Statement

The authors report no conflict of interest.
